# A Wearable Multimodal Wireless Sensing System for Respiratory Monitoring and Analysis

**DOI:** 10.3390/s23156790

**Published:** 2023-07-29

**Authors:** Kee S. Moon, Sung Q Lee

**Affiliations:** 1 Department of Mechanical Engineering, San Diego State University, San Diego, CA 92182, USA; 2 Electronics and Telecommunications Research Institute, Daejeon 34129, Republic of Korea

**Keywords:** biomedical signal processing, wearable biomedical sensors, medical equipment, multi-sensor fusion, respiration, digital health

## Abstract

Wireless sensing systems are required for continuous health monitoring and data collection. It allows for patient data collection in real time rather than through time-consuming and expensive hospital or lab visits. This technology employs wearable sensors, signal processing, and wireless data transfer to remotely monitor patients’ health. The research offers a novel approach to providing primary diagnostics remotely with a digital health system for monitoring pulmonary health status using a multimodal wireless sensor device. The technology uses a compact wearable with new integration of acoustics and biopotentials sensors to monitor cardiovascular and respiratory activity to provide comprehensive and fast health status monitoring. Furthermore, the small wearable sensor size may stick to human skin and record heart and lung activities to monitor respiratory health. This paper proposes a sensor data fusion method of lung sounds and cardiograms for potential real-time respiration pattern diagnostics, including respiratory episodes like low tidal volume and coughing. With a *p*-value of 0.003 for sound signals and 0.004 for electrocardiogram (ECG), preliminary tests demonstrated that it was possible to detect shallow breathing and coughing at a meaningful level.

## 1. Introduction

Recent advances in sensors for environmental and physiological monitoring, signal processing, and machine learning offer a significant opportunity for improving the quality of life for people living with chronic diseases while also providing new insights to the research community [1,2]. To analyze, treat, and reduce adverse health events, synchronized devices such as smartphones, tablets, and laptops will be necessary to connect data and devices and establish closed-loop or human-in-the-loop systems. Intelligent wearable sensors have the advantage of being able to be monitored and diagnosed anywhere because they are powered by a small battery, as opposed to typical laboratory equipment, which is generally constrained to a specific area connecting to a power outlet. Intelligent health monitoring systems necessitate the processing of high-resolution, multidimensional biological/medical data in real time. The advantage of user-dependent personal health models is that they record each user’s unique behavior, which typically results in a more accurate diagnosis [1,2].

Prior to the coronavirus epidemic, chronic respiratory disorders were America’s third most prominent cause of mortality [3,4]. Asthma, for example, is one of the most common chronic disorders in the United States, affecting 18.7 million adults (8%) and 6.8 million children (9.3%) [3,4]. Clinic visits are typically the primary setting for physicians to examine the current degree of asthma control and identify potential environmental triggers. Medication adherence has improved with integrated treatment models that include individualized self-management and shared treatment decision making between patients and physicians [5]. For example, a remote method for identifying asthma triggers and exacerbating factors is essential for personalized medicine. Individualized treatment plans and shared decision making have improved patient adherence and outcomes [6].

This study describes our continuing research toward constructing a wearable multimodal wireless sensing device for respiratory monitoring and analysis. The real-time wearable device offers a comprehensive solution for remotely monitoring pulmonary physiological responses. In this study, a small sensing device in an individual’s chest measures respiratory episodes such as coughing and vitals such as heart rate and respiratory rate. Furthermore, the machine-learning technique is utilized to detect respiratory occurrences that can be used to classify the severity of respiration-related symptoms in the future.

Several research groups have published findings on various techniques of monitoring respiration. Several research studies connected inertial measurement units to the chest and belly to observe breathing patterns [7,8]. A low-power nanosensor, such as a humidity sensor, has been published for monitoring respiratory patterns [9]. Furthermore, soft electronics applied to the skin have recently been claimed to detect human motions as breathing patterns [10]. Precisely measuring tidal volume helps in the diagnosis of respiration. Lung capacity has also been assessed using bioimpedance tools [11]. Previously, Moon et al. suggested the tidal volume sensors by combining sound and electromyogram (EMG) from the diaphragm muscle location [12]. Although this method works well for measuring continuous respiratory volume, signals can only be picked up close to breathing-related muscles, like the external intercostal muscles.

One of the oldest and most popular ways to identify early respiratory disorders with abnormal lung sounds is to understand the features of breathing sounds. It is also generally understood that breathing cycles and heartbeats are intricately related [13,14]. A respiratory signal can be obtained from an ECG (electrocardiogram) using a variety of approaches [15,16,17,18,19,20]. ECG variations analysis, for example, can be used to quantify respiratory motion. Another well-known method is based on observing the beat-to-beat fluctuations in RR intervals or their reciprocals, which, in most people, are caused by respiratory sinus arrhythmia (RSA). This approach works best in young, healthy patients with pronounced RSA. Furthermore, during inhaling, the heart rate rises and decreases [21].

This paper offers a novel method for remotely monitoring lung health with a wearable and integrated ECG and sound sensing method to detect changes in lung function based on the previous clues in detecting respiration through each signal of ECG and sound. As a result, combining respiratory sounds and cardiovascular physiological effects can help in more precisely modeling respiration episode patterns such as shortness of breath, coughing, and so on.

Wearability, for example, has been enhanced by substantial advances in materials, construction, and integration techniques. However, in order to construct truly wearable sensors that are both practical and clinically meaningful, we must overcome technical difficulties, in particular, continuous respiratory monitoring, such as battery power sources, sensor data storage, wireless data exchange, and automated diagnosis [1].

The following details how this paper is structured. Section 2 describes a custom-designed multi-channel integrated sensor system for simultaneously sensing lung sound and heartbeats utilizing a sound transducer and a set of ECG electrodes while comfortably adhering to human skin. Section 3 outlines the work that developed indices to assess lung functioning using mathematical methods. Finally, Section 4 examines the new signal processing technology suitable for sensor fusion and prospective machine-learning methods. The novel approach can incorporate sensor data of different dependability and sensitivity.

## 2. Wearable Lung Health Monitoring System and Experimental Setup

**Figure 1:**
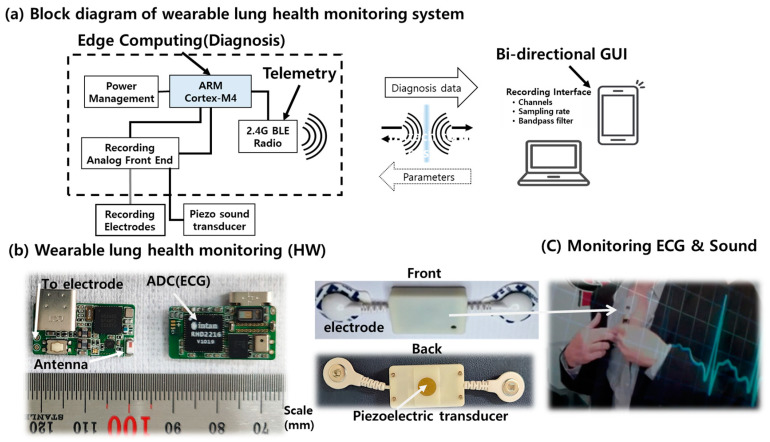
The wireless wearable respiratory health monitoring system.

Combining a piezoelectric sound sensor and an electrode-integrated ECG sensor, we designed and manufactured a unique wearable lung health monitoring system that will monitor cardiovascular and respiratory activities to deliver a quick digital diagnosis of lung health status (Figure 1 and Table 1). The wearable wireless system uses acoustics and biopotentials to monitor heart valve sounds, lung motions, and ECGs at the same time [22]. Furthermore, the wearable sensor system hardware is designed in a four-layer printed integrated circuit board using a software package (Mentor Graphics Co., Wilsonville, OR, USA, Board Station EN2004). One system was built on systems-on-chips (SoC) and systems-on-module architectures to avoid component interference and to operate on a hierarchical basis for a compact form factor, with a signal-processing circuit that performs edge computing prior to data transfer via a wireless data transmission chip. The transmitted signals are evaluated and diagnosed using an external computer system. The result of signal processing in the external computer system is the feature categorization result for clinical diagnostic decision-making, such as tidal volume in normal respiration and abnormal coughing.

A piezoelectric transducer converts sound signals from the heart and lungs into analog electrical signals. In addition, it facilitates automatic acoustic interpretation in diagnostics for the cardiovascular and respiratory systems. The sound acquisition module employs a piezoelectric microphone with a 4 kHz sampling rate. Inside the wearable sensor unit, the digital electrophysiology interface chips from Intan Technologies, LLC (Los Angeles, CA, USA) are used for the signal acquisition module. The RHD2216 chip, a low-power 16-channel differential amplifier integrated with a 16-bit analog-to-digital converter (ADC), was used to record the ECG and sound signal. The wearable sensor uses Bluetooth low-energy (BLE) technology for wireless communication. The signal is then wirelessly transmitted to a personal computer (PC), where MATLAB is utilized to classify and process the signal data. Multiple sensor signal acquisitions, amplification, filtering, digitization, and wireless transmission are performed in real time by the system. Because of its low power consumption and flexibility, we chose the Nordic Semiconductor nRF52832 System-on-Chip (SoC) for computing and wireless data transmission on the module. As an electrode for ECG, two commercially available adhesive ECG patches, a custom wearable sensor was designed and implemented for this investigation, as shown in Figure 1.

To optimize patient care, it is suggested that patients monitor their symptoms and respiratory function at home, which necessitates remote management of their condition. Currently, spirometry is frequently used for asthma diagnosis and monitoring, and a significant proportion of patients continue to have inadequate asthma control. A rapid diagnosis and effective treatment can reduce the burden of asthma-related illness. Developing accurate, efficient, and user-friendly asthma monitoring technologies is essential to achieving a more intelligent sensor technique, although traditional spirometry remains the most precise method for assessing lung function and airflow restriction. However, the equipment is large and requires close supervision.

As shown in Figure 1, a wearable sensor system was implemented for this investigation. During data collection at the described sensor placement, an experimental protocol was followed. Using integrated acoustics and biopotentials to detect heart and breathing activity, the technique can provide the ability to monitor symptoms in a novel manner. The sensor adheres to the skin, similar to a small bandage. It continuously monitors heart and lung function.

A set of experimental sensor signals was collected from two healthy males with slow, constant-rate respiration (i.e., 2 s inhale and 2 s exhale) for this study. All of the investigations were carried out while the subjects were seated in a quiet place. The breathing cycle was controlled using a metronome (https://www.imusic-school.com/en/tools/online-metronome/, accessed on 31 December 2020). Three different tidal volumes (i.e., 1000, 750, and 500 mL) were controlled using a Voldyne 5000 Spirometer (Hudson RCI, calibrated with PF100 digital Peak Flow and FEV1 Meter, Microlife, Clearwater, FL, USA). The wearable system has a bandpass filter program installed to maximize the sensor’s heart and lung signals. The sampling frequency was set at 4 kHz per channel for the experiment.

Table 2 lists the experiments conducted. Experiment I depicts a deep breathing pattern (1000 mL tidal volume) with slow, constant-rate respiration (i.e., 2 s inhale and 2 s exhale). Experiment II depicts moderate breathing with the same respiration rate, and Experiment III demonstrates shallow breathing with a 500 mL volume. Experiment IV also has a pattern of coughing that simulates an episode of asthma coughing.

**Figure 2:**
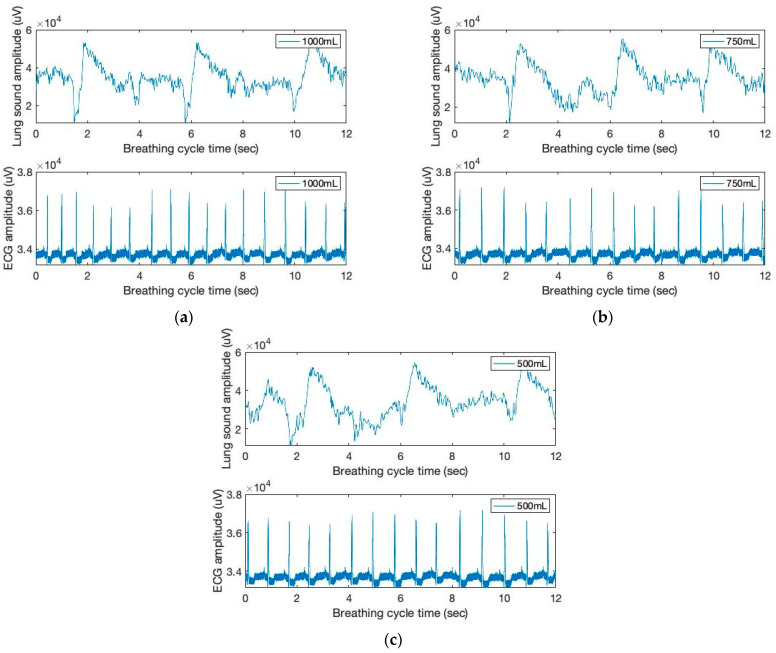
The time and amplitude characteristics of lung sound and ECG signals of three breathing cycles obtained from the chest location; (**a**) 1000 mL, (**b**) 750 mL, and (**c**) 500 mL tidal volumes; one breathing cycle: 2 s inhale and 2 s exhale.

Figure 2 depicts the time and amplitude characteristics of a typical lung sound and ECG signal obtained from the chest area. The figure shows the breathing cycles of the inhale and the following exhale phases. Due to the close coupling between respiration cycles and heartbeats, the sound and ECG signals in the figure exhibit synchronized patterns on the graphs. [R] In addition, the height variations of the heartbeat signal during inhale and exhale are visible in the ECG signal graph (bottom). Further, during the inhale phase, the heartbeat signal heights are known to decrease [21].

## 3. Modeling of Respiration Function

### 3.1. Sound Signal Modeling

**Figure 3:**
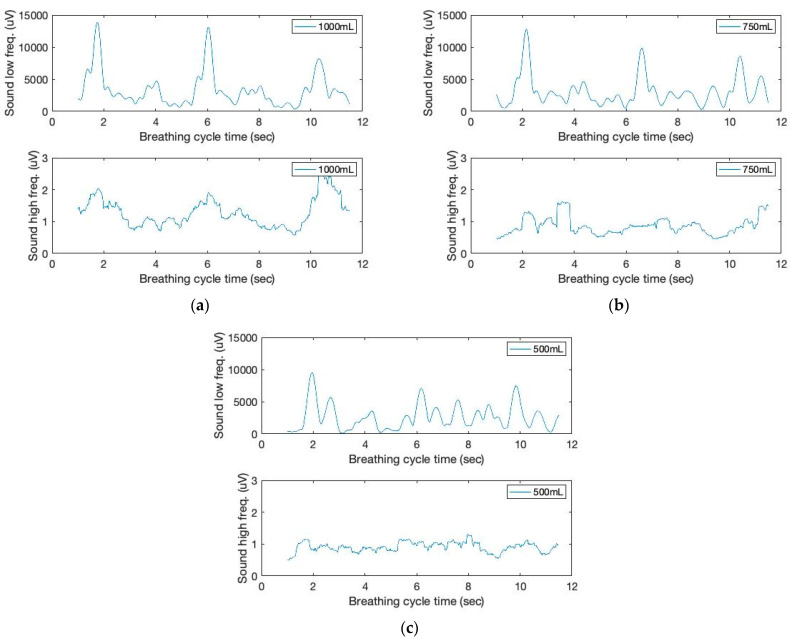
The time and filtered amplitude characteristics of lung sound signals of three breathing cycles obtained from the chest location; (**a**) 1000 mL, (**b**) 750 mL, and (**c**) 500 mL tidal volumes; one breathing cycle: 2 s inhale and 2 s exhale.

Various methods have been available for the acoustic sound monitoring of patient breathing. For example, on occasion, aberrant lung sounds, such as wheezing or stridor, can be detected at frequencies greater than 2000 Hz [23,24,25,26]. This study classifies lung sounds for sound signal filtering using low (below 2 Hz) and high (above 150 Hz) frequency ranges, respectively. The filtered signals are shown in Figure 3. Piezo sound transducer can detect low-frequency movement as well as high-frequency sound. Noteworthy is the fact that the low-frequency sound signal can provide inhalation peaks, which exhibit a higher signal intensity with increased tidal volumes. Low frequency, on the other hand, is not repetitive to the contact force of the sound sensor on the skin. Instead, we make use of high-frequency sound signals that exhibit increased intensity and more distinct matching peaks during the inhalation cycles.

**Figure 4:**
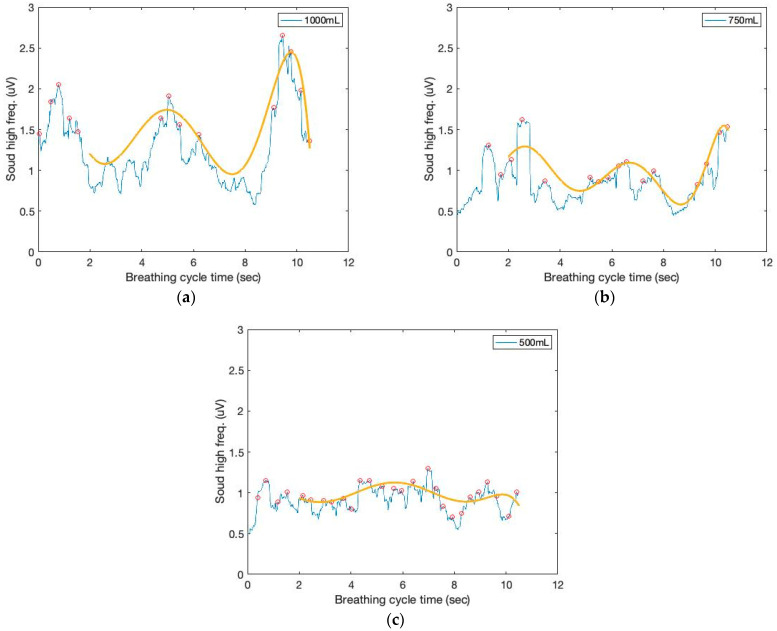
The time and filtered amplitude characteristics of the high-frequency filtered signals and a seventh-order polynomial curve fit of three breathing cycles obtained from the chest location; (**a**) 1000 mL, (**b**) 750 mL, and (**c**) 500 mL tidal volumes; one breathing cycle: 2 s inhale and 2 s exhale.

**Figure 5:**
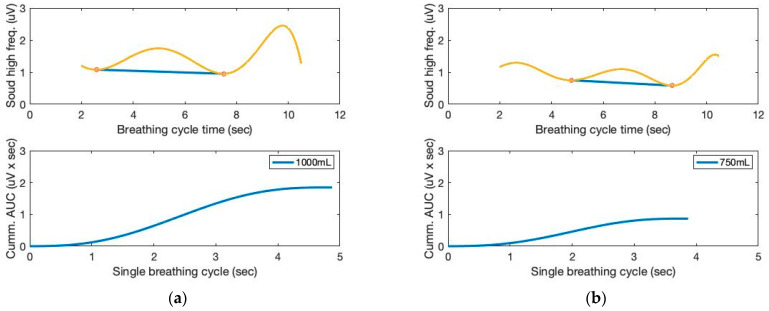

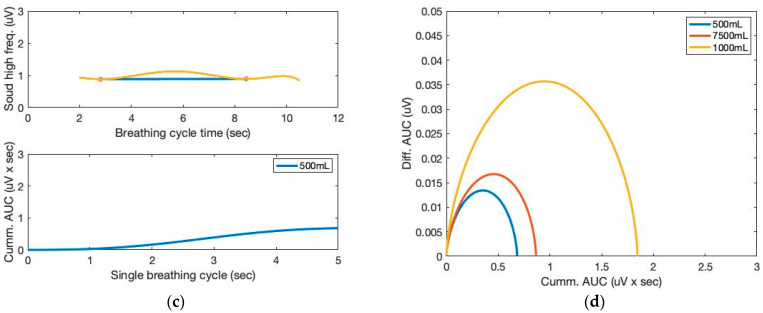
The AUC and cumulative AUC from lung sound signals obtained from a seventh-order polynomial curve fit of three breathing cycles obtained from the chest location; (**a**) 1000 mL, (**b**) 750 mL, and (**c**) 500 mL tidal volumes; one breathing cycle: 2 s inhale and 2 s exhale. (**d**) The cumulative derivative AUCs relate to lung volume during inhaling.

Moving variances are also calculated with a 250 ms data window and 1000 samples. The moving window variance computes the variability of each window’s sensor signal. Figure 4 depicts the high-frequency filtered signals and a seventh-order polynomial curve fit derived from the proposed sound sensor to separate low-frequency lung sound signals from the filtered signals. Identifying the repetition of the characteristic patterns and the peak heights from the cyclic pattern of the polynomial graphs enables monitoring respiration cycles and tidal volumes from the area under curve (AUC). The AUC calculated from the high-frequency filtered signals and a seventh-order polynomial curve fit are depicted in Figure 5. The AUCs derived from the sound sensor make approximately estimating tidal volumes possible without a spirometer. We calculated the derivative of the cumulative AUCs. The cumulative derivative AUCs relate to lung volume during inhaling. It demonstrates the different tidal volume patterns, which may be used for evaluating breathing conditions.

### 3.2. ECG Signal Modeling

**Figure 6:**
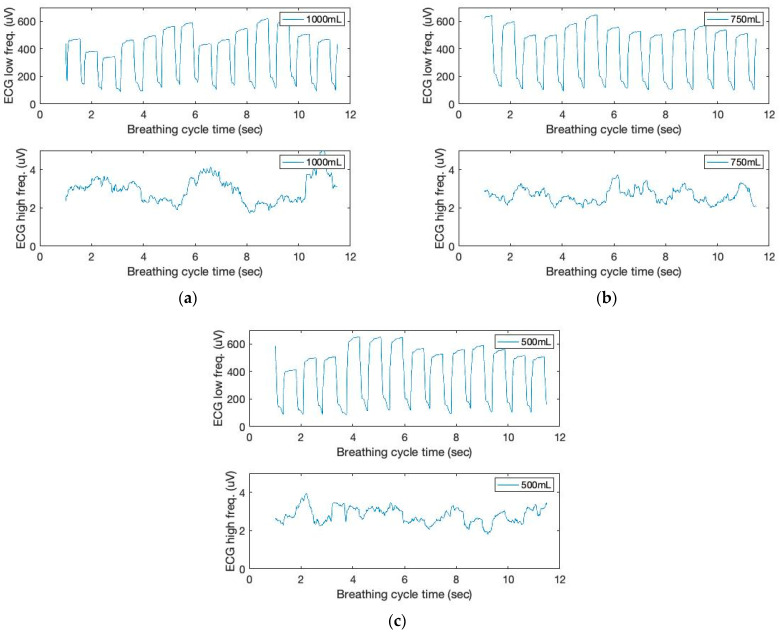
The time and filtered amplitude characteristics of ECG sound signals of three breathing cycles obtained from the chest location; (**a**) 1000 mL, (**b**) 750 mL, and (**c**) 500 mL tidal volumes; one breathing cycle: 2 s inhale and 2 s exhale.

As shown in Figure 2, the respiratory and cardiac cycles are interconnected. This study uses low- (below 150 Hz) and high-frequency ranges (above 150 Hz) to filter ECG signals. In addition, moving variances are computed using a 250 ms data window and 1000 samples. The moving window variance calculates the ECG sensor signal variation for each window. Figure 6 depicts the filtered signal outputs. From the graph, we can see that the peak height varies from beat to beat. Clearly, typical pulse signals are synchronized with respiration phases, with signal heights decreasing during inhale and increasing during exhale.

**Figure 7:**
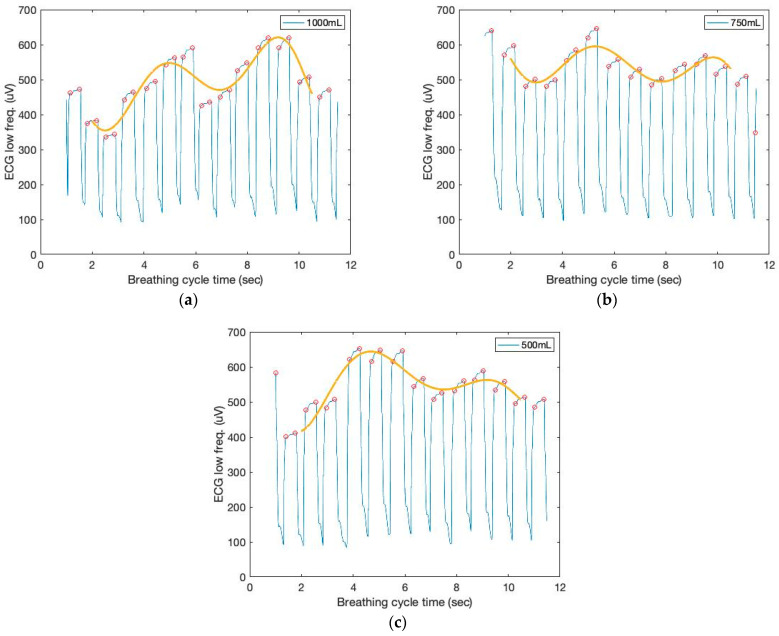
The time and filtered amplitude characteristics of the low-frequency filtered ECG signals and a seventh-order polynomial curve fit of three breathing cycles obtained from the chest location; (**a**) 1000 mL, (**b**) 750 mL, and (**c**) 500 mL tidal volumes; one breathing cycle: 2 s inhale and 2 s exhale.

Figure 7 depicts the low-frequency filtered signals and a seventh-order polynomial curve fit derived from the ECG sensor to identify the repetition of the characteristic patterns and the peak heights from the cyclic pattern of the polynomial graphs, which enables the monitoring of respiration cycles and tidal volumes based on the AUC.

**Figure 8:**
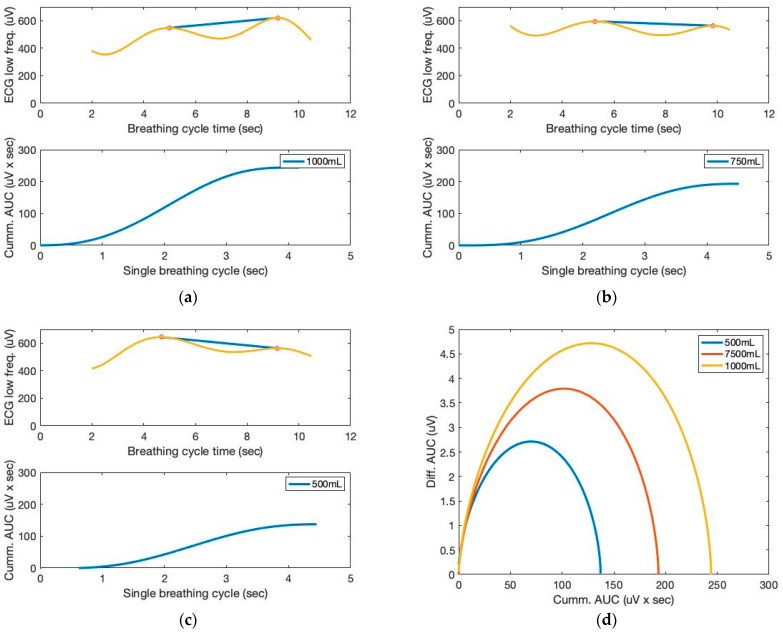
The AUC and cumulative AUC from ECG signals obtained from a seventh-order polynomial curve fit of three breathing cycles obtained from the chest location; (**a**) 1000 mL, (**b**) 750 mL, and (**c**) 500 mL tidal volumes; one breathing cycle: 2 s inhale and 2 s exhale. (**d**) The cumulative derivative AUCs relate to lung volume during inhaling.

**Figure 9:**
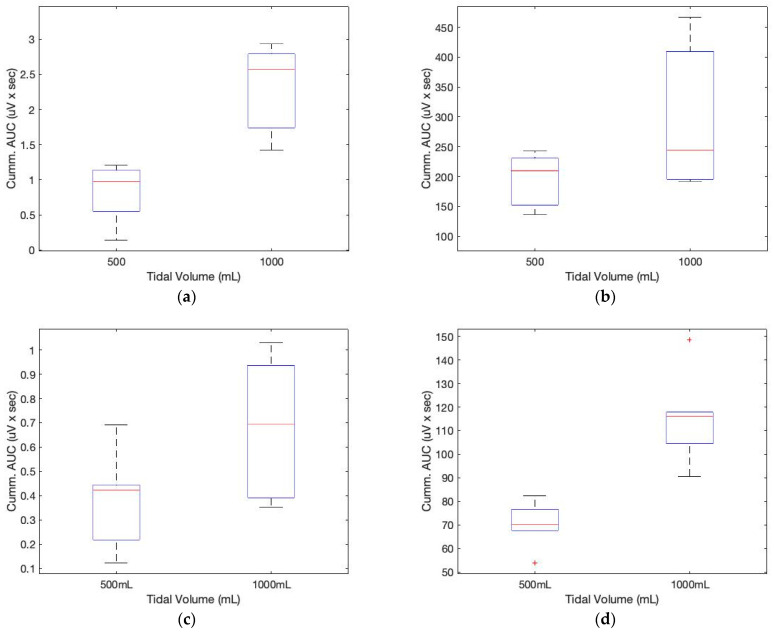
Box plots for the cumulative AUCs during one breathing cycle for different tidal volumes: (**a**) sound sensor data (subject 1); (**b**) ECG sensor data (subject 1); (**c**) sound sensor data (subject 2); (**d**) ECG sensor data (subject 2).

Figure 8 depicts the AUCs calculated from the low-frequency filtered signals and a seventh-order polynomial curve fit. Similar to the sound sensor signals, the AUCs derived from the ECG sensor also allow for the approximation of the tidal volume. The cumulative derivative AUCs relate to lung volume during inhaling. Measurements of tidal volume can be used to differentiate abnormal respiratory patterns. The statistical analysis of box plots and the F-test contribute to the experimental findings. Figure 9 depicts the box plots for the AUCs (five breathing cycles) derived for the various data set groups of 1000 mL and 500 mL tidal volumes, respectively.

**Figure 10:**
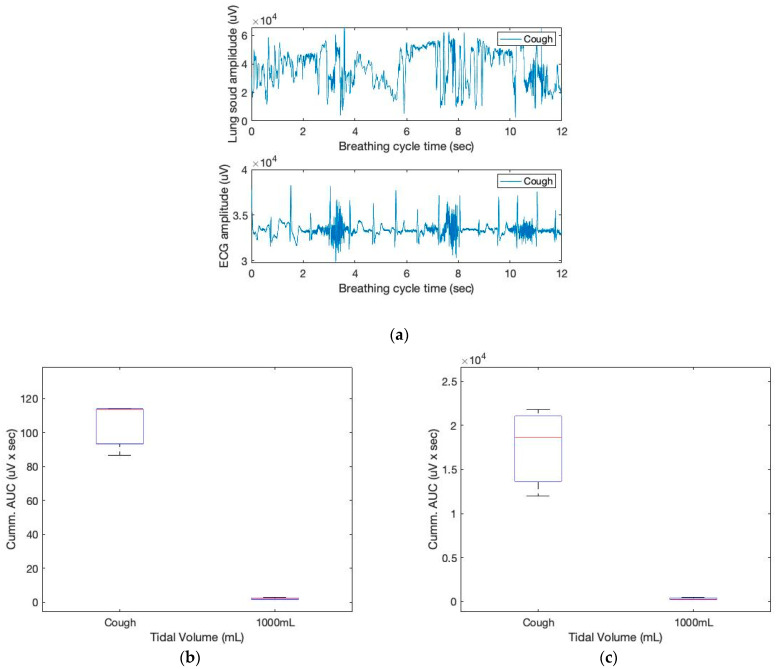
The sound and ECG signals of three coughing cycles obtained from the chest location (**a**); box plots for the cumulative AUCs during one breathing cycle for different tidal volumes: (**b**) sound sensor data; (**c**) ECG sensor data.

When something irritates the airways or larynx, the body reacts by coughing. Deep inhalation, glottic closure, the contraction of the respiratory muscles with glottic opening, and intercostal and abdominal muscle relaxation are the phases of a cough. In this study, we simulated coughing to comprehend the sensor responses. Figure 10 depicts the sound and ECG sensor signals during the breathing episodes. In addition, the figure’s box diagrams provide a strong indication of deep inhalation with increased tidal volume.

In Table 3, the F-value calculation represents the ratio of group variation to within-group variation. A high F-value indicates that the variation between groups is greater than the variation within groups. This indicates that there is a statistically significant difference between the means of our groups. In the table, the values in parentheses indicate the F and *p* values of subject 2. The F critical value is a specific value against which the F-value can be compared to ascertain the statistical difference. From the table and Figure 9, it is evident that the calculated F value for both the sound and ECG signals is greater than the F critical value, indicating a difference in the cumulative AUC for the various digital volumes. In addition, the computed *p*-values are smaller than the 0.05 value used in practice. The F statistic determines the *p*-value, which is the probability that the test results could have occurred by coincidence.

## 4. Lung Sound and ECG Sensor Signal Fusion

People who have breathing difficulties frequently exhibit symptoms of respiratory distress, which includes having to work harder to breathe or not receiving enough oxygen. For instance, chronic airway inflammation known as asthma causes reversible, episodic airway restriction and is frequently accompanied by wheezing, coughing, crackling, and gasping for air. Auscultation, or the listening to and classification of a body’s internal noises, has traditionally required the use of a stethoscope by a qualified expert. The use of electret microphones and piezoelectric sensors for auscultation as well as various sensing approaches for categorization have, however, been the subject of recent research [27,28,29,30,31,32,33,34,35,36]. Measures of lung health can be obtained from respiratory activities. The ability to continuously characterize respiratory activities, frequently needed to assess respiratory illnesses and diseases, needs to be improved via current approaches. Efforts have described a machine-learning algorithm-equipped device that can continually track breathing activities [37,38,39].

In this study, an integrated sensor fusion and associated analysis method are presented to enable ubiquitous respiratory episode monitoring. Monitoring and analyzing respiration data can provide vital clinical information to healthcare consumers and the public. Assessing breathing patterns based on respiratory episodes such as shallow breathing and coughing, for instance, enables the early recognition of asthma exacerbation symptoms. We utilized a two-sensor system to overcome the limitations of monitoring tidal volumes and respiration patterns using a single sensor-based measurement. This work contributes to the classification of distinctive respiration patterns based on sound and ECG signals. The following procedures were taken to identify the specific breathing patterns of interest:

- Determining the cumulative AUC from lung sound and ECG signals using a seventh-order polynomial curve fit;
- Transformation of the AUCs into a series of signature matrices;
- Classification of the signature matrices’ characteristic respiration patterns.

Using signature matrices, this research presents a novel method for classifying patterns for sensor fusion of the sound and ECG signals [22]. The calculated cumulative AUCs are used to generate three-dimensional signature matrix patterns. For example, respiration symptom classification templates can be derived from a well-planned series of experiments that capture the characteristic (or signature) behavior of respiration disorders. Utilizing a probability map, the numeric values of this signature matrix represent compressed respiration pattern characteristics. In this study, the elements of the signature matrix represent the probability that the cumulative AUCs at a sampling point meet a specific classification criterion. For instance, to compute the probability map, a frequency count can be derived from the lower, middle, and upper values of cumulative AUCs calculated from typical lung sounds and ECG signals, respectively. From the sensor signals, a signature matrix generates a series of two-dimensional “image-like” signature matrix patterns. These signature matrix patterns reflect the compressed characteristics per sampling window using the probabilities as image frame pixels. The classification zone can be constructed from a set of well-planned subgroups (i.e., a template) in order to estimate the signature (or characteristic) behavior of a sensor signal pattern’s means.

A [3 × 3] two-dimensional signature matrix was rearranged in this study as a [3 × 3] matrix-sized probability map. For two sensors, a signature matrix (i.e., frame) *SIG_k_* for sample point k (i.e., each breathing cycle) is defined by the following equation:

(1) $${SIG}_{}^{k}=\left[\begin{array}{ccc} {sig}_{11} & {sig}_{12} & .\\ {sig}_{21} & {sig}_{22} & .\\ . & . & {sig}_{33} \end{array}\right]\;$$

For a selected combination of sample sensor signals (say, channels *X* and *Y*), a frequency count can be derived from a matching column *j* and row *i* for the corresponding zone of channels *X* and *Y*. Consequently, a signature matrix is a probability map depicting the likelihood that a selected sample belongs to a particular classification zone, which is expressed as follows:

(2) $${sig}_{ij}=P\left[\begin{array}{c} \left(a\;mean\;of\;X\;belongs\;to\;class\;j\;\right)\\ ⋂\left(a\;mean\;of\;Y\;belongs\;to\;class\;i\;\right) \end{array}\right]$$

In this study, the signature matrix can be described as a two-dimensional probability diagram (or probability map) with a 3 × 3 matrix size. The method uses a two-dimensional signature matrix pattern from Equation (2). The signature matrices used for high and low cumulative AUCs were obtained from the sound and ECG sensor signals. The data were collected in a sampling size of 40 data points. Thus, there will be 100 samples per second if the sampling frequency is 4 kHz, the sampling frequency used for the lung sensor signal monitoring. These signature matrix patterns represent the compressed characteristics of sensor signals for a given data length of interest. Comparing the measured signature matrix patterns (i.e., targets) with the standard patterns (i.e., templates) enables a real-time sensor data assessment. The method begins by separating the cumulative AUCs into zones. Using a preset interval, this investigation classified and assigned sound and ECG signals to their corresponding pixels. The following are the calculated 3 × 3 template signature matrices for a set of selected samples.

(3) $${SIG}_{1000\mathrm{mL}(1)}^{k}=\left[\begin{array}{ccc} 0.0 & 0.0 & 0.2421\\ 0.0 & 0.5263 & 0.0\\ 0.2211 & 0.0 & 0.0 \end{array}\right]$$

(4) $${SIG}_{500\mathrm{mL}(1)}^{k}=\left[\begin{array}{ccc} 0.0 & 0.0 & 0.0\\ 0.0 & 0.7053 & 0.0\\ 0.2842 & 0.0 & 0.0 \end{array}\right]$$

(5) $${SIG}_{cough(1)}^{k}=\left[\begin{array}{ccc} 0.0 & 0.0 & 0.9053\\ 0.0 & 0.0105 & 0.0737\\ 0.0 & 0.0 & 0.0 \end{array}\right]$$

(6) $${SIG}_{1000\mathrm{mL}(2)}^{k}=\left[\begin{array}{ccc} 0.0 & 0.0211 & 0.3474\\ 0.0 & 0.2421 & 0.0\\ 0.1474 & 0.2316 & 0.0 \end{array}\right]$$

(7) $${SIG}_{500\mathrm{mL}(2)}^{k}=\left[\begin{array}{ccc} 0.0 & 0.0 & 0.0\\ 0.0 & 0.5158 & 0.0\\ 0.4 & 0.0737 & 0.0 \end{array}\right]$$

A periodical diagnosis can be conducted from the sensor data assessment by comparing the measured signature matrix patterns (i.e., targets) with the stored template patterns. The squared difference between the template pixel and the measured pixel is provided by the following:

(8) $$d_{ij}^{k}={\left({{sig}_{ij}^{T}-sig}_{ij}^{k}\right)}^{2}$$

(9) $${SIG}_{template(1)}^{T}=\left[\begin{array}{ccc} 0.0 & 0.0631 & 0.0807\\ 0.0035 & 0.5438 & 0.0631\\ 0.2281 & 0.007 & 0.0 \end{array}\right]$$

(10) $${SIG}_{template(2)}^{T}=\left[\begin{array}{ccc} 0.0 & 0.0105 & 0.4105\\ 0.0 & 0.2211 & 0.0\\ 0.1474 & 0.2 & 0.0 \end{array}\right]$$

The sum of squared differences at the sampling period *k* can be calculated to observe the errors between the pre-stored template signature matrices and the measured matrices.

(11) $$E_{ij}^{k}=\sum_{i=1}^{N}\sum_{j=1}^{N}d_{ij}^{k}$$

**Figure 11:**
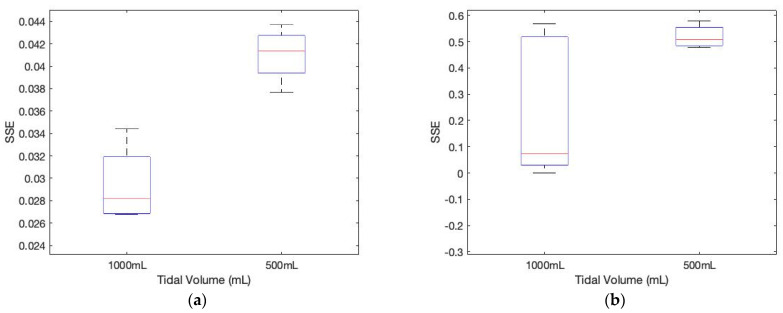
The sum of squared errors between the pre-stored template signature matrices and the measured matrices for different tidal volumes. (**a**) subject 1 (*p*-value: 0.0018); (**b**) subject 2 (*p*-value: 0.052).

As shown in Figure 11, the sum of squared errors between the pre-stored template signature matrices and the measured matrices for different tidal volumes demonstrates the different tidal volume patterns, including coughing, which may be used for evaluating breathing conditions.

In this study, the proposed method demonstrates that the cumulative AUCs calculated from the lung sound and heart ECG sensors from the chest area exhibit discernible patterns, as shown in Equations (3)–(7). In identifying aberrant respiratory patterns from a signature matrix, the matrix elements in the equations demonstrate the changes between normal (i.e., template or 1000 mL) and abnormal (i.e., target or 500 mL and coughing) during breathing cycles. The essential indicator of this method is the recognition of shallow breathing, which is one of the most challenging tasks for wearable sensors. Incorporating the signature matrices for the training data set and the input layers, this study demonstrates the prospective integration capability of a standard artificial neural network (ANN) as a feature extractor. During model training, there is no need to normalize the training data set from the various sensors, such as the noises and the heartbeats, within the elements of the signature matrices.

Prior research suggests that spirometry needs to be more utilized to diagnose asthma for diagnosis and follow up [40]. However, due to the difficulty of using the spirometer continuously, especially at night, there is a strong need for wearable technology that can supplement conventional spirometry for respiratory episode monitoring and diagnosis. The marginalized population with difficulty visiting hospitals and receiving care from a specialist for diagnosis and follow up would benefit from wearable technology. The proposed technology would require more stringent medical testing with more precise calibration procedures that correct the sensitivity variations in future applications to a population of patients receiving medical care.

## 5. Conclusions

Accessibility of human health data is a crucial concept for the future culture and success of biology, as the democratization of data is essential for attracting talent to concentrate on the complex problems posed by the inherent complexity of biological systems. This will be even more important in the medical field, where scientists will need access to the data cloud available from each individual to mine for future predictive treatments. Reliable and remote monitoring of health conditions is essential for critically ailing patients as well as healthy individuals. To develop truly practical and clinically significant wearable sensors, however, we must first surmount a number of technical obstacles.

For instance, machine-learning techniques are frequently used to analyze sensor data because they are well-suited for processing the enormous amount of data generated by wearable sensors and detecting specific patterns and mutations associated with various diseases. Spirometry is frequently underutilized for the diagnosis and monitoring of respiratory symptoms. Nevertheless, their real-time prediction performances of spirometry-based monitoring still need to be closer to the level of wearability required for practical use. In this study, we demonstrated that the multimodal wearable sensor system can detect abnormal lung sounds caused by respiratory problems, such as shallow breathing or coughing. Further, it also proposed utilizing ECG signals to assess respiratory functions in addition to cardiovascular issues.

This paper describes a novel method for identifying lung functions by combining two distinct categories of sensor signals using a signature matrix. Moreover, the approach is not only suitable for the integration of multimodal sensory data, but it can also aid in the development of machine-learning models that classify sensor signals to determine specific symptoms.

## 6. Patents

The following patent partially results from the work reported in this manuscript: K. Moon, S. Lee, and W. Youm, “An interactive health-monitoring platform for wearable wireless sensor systems”, 2021. PCT/US20/51136.

## Figures and Tables

**Table 1:** The specifications of the wireless wearable health sensor.

Specification	Description	Value	Comments
Sound transducer	piezoelectric plate	10 mm diameter	Sound from lung
ECG electrodes	Disposable Ag/AgCl standard pre-gelled and self-adhesive	(20 × 20) mm	Low impedance pre-gelled electrode
Front-end circuit	Intan Tech Chip	10 mV, 16 bit, 8 ch	High resolution and low noise
Onboard CPU	ARM Cortex M4	4096 Hz sampling rate, onboard computing	Real-time data processing
Wireless Data transmission	NRF 52X, BLE5.0	2.4 GHz Carrier, 1 Mbps data rate in 2 m distance	Enable Wearable service
Power source	rechargeable battery	8 h/charging	Internal battery for daytime

**Table 2:** A breathing cycle experimental protocol.

Experiment	Tidal Volume	Breathing Cycle
I—Deep breathing	1000 mL	4 s
II—Moderate breathing	750 mL	4 s
III—Shallow breathing	500 mL	4 s
IV—Coughing	Over 1000 mL	4 s

**Table 3:** An F-value and *p*-value table for cumulative AUC obtained from sound and ECG.

Tidal Volume	F-Value (Sound)	*p*-Value (Sound)	F-Value (ECG)	*p*-Value (ECG)
1000 mL vs. 500 mL	18.22 (4.11)	0.003 (0.07)	3.04 (26.86)	0.12 (0.0004)
1000 mL vs. Cough	128.81	0.003	34.93	0.004
Critical value	5.318 (4.965)	0.05 (0.05)	5.318 (4.965)	0.05 (0.05)

## Data Availability

Not applicable.
